# Disruption and Adaptation of Antimicrobial Stewardship Programs in Iranian Hospitals During the COVID-19 Pandemic: A Descriptive Phenomenological Qualitative Study

**DOI:** 10.7759/cureus.88754

**Published:** 2025-07-25

**Authors:** Behzad Bijani, Laleh Azizi, Reza QasemiBarqi, Abbas Allami

**Affiliations:** 1 Infectious Diseases, BuAliSina Hospital, Qazvin, IRN; 2 Infectious Diseases, Medical Microbiology Research Center, Qazvin University of Medical Sciences, Qazvin, IRN; 3 Infectious Disease Specialist, Alborz University of Medical Sciences, Karaj, IRN; 4 Infectious Disease, BuAliSina Hospital, Qazvin, IRN

**Keywords:** antibiotic resistance, antimicrobial stewardship, covid-19, iran, qualitative study

## Abstract

Introduction

Antimicrobial resistance (AMR) is a pressing global health issue, exacerbated by inappropriate antibiotic use. In Iran, the 2016 National Action Plan on AMR established hospital-based antimicrobial stewardship programs (ASPs), including audit-feedback, prescription restrictions, and guideline-based prescribing to promote rational prescribing. The COVID-19 pandemic disrupted these efforts, resulting in increased empirical antibiotic use. This qualitative study, using a descriptive phenomenological approach, explores healthcare providers’ perceptions of antibiotic prescribing and ASP implementation in two Iranian teaching hospitals during the COVID-19 pandemic, focusing on context-specific barriers and adaptations.

Methods

Conducted from May 20, 2022, to January 20, 2023, at two 500-bed teaching hospitals in Qazvin, Iran, this descriptive phenomenological study involved 12 purposively sampled healthcare providers (10 physicians, one clinical pharmacist, one microbiology lab supervisor) until thematic saturation, achieved when no new themes emerged after the 10th interview, as confirmed by codebook review. Semi-structured interviews (45-60 minutes), conducted in Persian in private settings, explored prescribing changes, ASP disruptions, and adaptations. Transcripts were translated into English, anonymized, and analyzed thematically, generating ~150 codes clustered into four themes. Trustworthiness was ensured via triangulation, audit trails, and member checking, with translation accuracy verified by a second bilingual researcher. Ethical approval was granted by the Qazvin University of Medical Sciences Ethics Committee (IR.QUMS.REC.1403.091).

Results

Four themes emerged from the qualitative analysis, reflecting participant-reported experiences: (1) Increased empirical prescribing - all participants reported diagnostic uncertainty and public pressure driving preemptive use of broad-spectrum antibiotics (e.g., ceftriaxone, meropenem). (2) Disruption of stewardship programs - most participants (10/12) noted suspended ASP activities (e.g., audits) due to staff redeployment. (3) Systemic and laboratory constraints - all participants highlighted delayed cultures and antibiotic shortages as barriers. (4) Adaptations and lessons learned - most participants (10/12) described late-2021 adaptations like virtual consultations. All findings reflect participant-reported experiences and perceptions, not objectively verified outcomes.

Conclusion

This qualitative study provides exploratory insights into healthcare providers’ perceptions of AMS disruptions in two Qazvin hospitals, with reported increases in empirical prescribing due to uncertainty and resource constraints. Participant-reported adaptations, such as virtual reviews, were noted, though their sustainability remains unevaluated. International comparisons with the UAE, UK, and India highlight shared challenges and the need for context-specific protocols and diagnostics. Findings are limited to participant perceptions and require further validation through mixed-methods research. Policymakers may consider exploratory strategies like virtual tools and public education, pending feasibility studies.

## Introduction

Antimicrobial resistance (AMR) poses a critical global health threat, driven by inappropriate antibiotic prescribing, a challenge exacerbated during the COVID-19 pandemic [1]. Rational prescribing-using the correct antibiotic, dose, and duration only when indicated-is central to the World Health Organization’s strategy to curb resistance [1]. In Iran, particularly in Qazvin’s teaching hospitals, pre-pandemic studies reported that a significant portion of antibiotic prescriptions lacked clear indications, with national consumption rivaling Europe’s combined average [2]. To address this, Iran’s 2016 National Action Plan on AMR established hospital-based antimicrobial stewardship programs (ASPs), which promote rational antibiotic use through audit-feedback and guideline-based prescribing, though implementation varied due to resource constraints and regional disparities [3]. At the university level, Qazvin’s two teaching hospitals maintained active ASPs, including regular audits and infectious disease consultations, limited by staffing and diagnostic capacity [4]. The COVID-19 pandemic disrupted these efforts, with global reports indicating increased empirical prescribing despite low bacterial co-infection rates (e.g., only 14% of hospitalized COVID-19 patients in Iran had confirmed co-infections, yet they received an average of 1.21 defined daily doses of antibiotics per patient-day) [5, 6]. International surveys noted suspended stewardship activities and preemptive prescribing during pandemic surges, compounded by media-driven pressures [7]. Despite these insights, there is a lack of qualitative understanding of how Iranian healthcare providers adapted ASP practices during this crisis. This qualitative study explores the perceptions and experiences of physicians, pharmacists, and laboratory staff in Qazvin’s two teaching hospitals, focusing on barriers to ASP implementation and participant-reported adaptive strategies to inform approaches for strengthening AMS in resource-limited settings.

## Materials and methods

Study design and setting

This qualitative study employed a descriptive phenomenological approach to capture healthcare providers’ lived experiences regarding antimicrobial stewardship programs (ASPs) during the COVID-19 pandemic, chosen for its ability to provide in-depth, contextual insights into perceptions and adaptations. Conducted from May 20, 2022, to January 20, 2023, the study was set in two 500-bed teaching hospitals affiliated with Qazvin University of Medical Sciences, Iran, selected for their established ASPs and high patient volumes during COVID-19 surges, managing hundreds of COVID-19 admissions monthly at peak periods. The study adhered to the Consolidated Criteria for Reporting Qualitative Research (COREQ) checklist [8].

Participants and sampling

Twelve participants were purposively sampled based on their involvement in antibiotic prescribing or diagnostics, ensuring representation of diverse roles (10 physicians, one clinical pharmacist, one microbiology laboratory supervisor) with direct ASP experience or impact. Participants included those with formal ASP roles (e.g., infectious disease specialists, pharmacists) and healthcare providers impacted by ASPs (e.g., residents). Recruitment and interviews spanned nine months (May 20, 2022, to January 20, 2023) due to logistical challenges, including limited staff availability during post-COVID recovery. The sample size of 12 was determined by thematic saturation, achieved when no new themes emerged after the 10th interview, as confirmed by reviewing the codebook and interview data, consistent with qualitative research standards. All participants had pandemic experience and ASP familiarity. Informed consent was obtained, and interviews were anonymized (e.g., “Physician 1-10,” “Pharmacist,” “Lab Supervisor”).

Data collection

In-depth semi-structured interviews used a piloted interview guide, tested with two volunteer physicians. Questions explored prescribing changes and ASP challenges, e.g., “How did COVID-19 alter your antibiotic prescribing practices?” and “What barriers did the ASP face during the pandemic?” All interviews were conducted in-person in private hospital offices from May 20, 2022, to January 20, 2023, with no virtual interviews due to logistical preferences. Twelve interviews, conducted in Persian by a trained qualitative researcher, lasted 45-60 minutes. Audio recordings were transcribed verbatim within 48-72 hours, with field notes capturing non-verbal cues.

Data analysis

Transcripts, originally in Persian, were translated into English by bilingual researchers, with accuracy verified by a second bilingual researcher comparing English transcripts against Persian originals, resolving discrepancies through discussion; back-translation was not used due to resource constraints. Researchers familiarized themselves with the data through multiple readings, identifying significant statements related to antibiotic use and stewardship. Thematic analysis was conducted inductively, with code development involving generating ~150 initial codes from significant statements, refined into categories and themes through iterative team discussions, ensuring alignment with participants’ lived experiences. Saturation was assessed by reviewing the codebook and interview data, confirming no new themes after the 10th interview. No software was used; coding was conducted manually by two researchers, resolving discrepancies via consensus. Trustworthiness was enhanced via an audit trail, investigator triangulation, and member checking with two participants. The full interview guide is available as supplementary materials (Appendix 1).

Ethical considerations

Participants provided informed consent, with confidentiality and voluntary participation assured. No compensation or incentives were provided to participants, ensuring voluntary participation. Identifiable information was removed, and findings were reported in aggregate. Participants could skip questions or withdraw without consequence. Ethical approval was obtained from the Qazvin University Ethics Committee (code: IR.QUMS.REC.1403.091).

## Results

The findings, based on 12 participants, reflect context-specific experiences in two Qazvin hospitals, with limitations in broader applicability due to the small sample size. All findings reflect participant-reported experiences and perceptions, not objectively verified outcomes. The study involved a diverse group of healthcare providers purposively sampled to capture a range of perspectives on antimicrobial stewardship programs (ASPs) during the COVID-19 pandemic. Participants included physicians with specialties in infectious diseases, anesthesiology, internal medicine, and pulmonology, as well as a clinical pharmacist and a microbiology laboratory supervisor, all from two 500-bed teaching hospitals in Qazvin, Iran. This selection ensured representation of roles directly involved in antibiotic prescribing and diagnostics, including both formal ASP leaders (e.g., infectious disease specialists, pharmacists) and healthcare providers impacted by ASPs (e.g., residents). Table 1 below presents the detailed characteristics of the 12 participants, including their age, gender, role, specialty, academic or work position, and years of experience.

**Table 1:** Characteristics of Participants

Participant ID	Age (years)	Gender	Role	Specialty	Academic/Work Position	Years of Experience
P1	47	Male	Physician	Infectious Diseases	Faculty Member	15
P2	60	Male	Physician	Infectious Diseases	Faculty Member	30
P3	35	Male	Physician	Infectious Diseases	Contract Staff	5
P4	50	Male	Physician	Anesthesiology	Faculty Member	14
P5	47	Male	Physician	Anesthesiology	Faculty Member	15
P6	40	Female	Physician	Internal Medicine	Contract Staff	10
P7	45	Female	Physician	Pulmonology	Faculty Member	15
P8	42	Female	Physician	Pulmonology	Faculty Member	10
P9	45	Male	Laboratory Supervisor	Microbiology	MSc, Supervisor	20
P10	45	Female	Pharmacist	Clinical Pharmacy	Head of Pharmacy	20
P11	30	Female	Resident	Internal Medicine	Resident	4
P12	35	Female	Resident	Internal Medicine	Resident	4

Four themes emerged from the qualitative analysis, reflecting participant-reported experiences: (1) Increased empirical prescribing - all participants reported diagnostic uncertainty and public pressure driving preemptive use of broad-spectrum antibiotics (e.g., ceftriaxone, meropenem). (2) Disruption of stewardship programs - most participants (10/12) noted suspended ASP activities (e.g., audits) due to staff redeployment. (3) Systemic and laboratory constraints - all participants highlighted delayed cultures and antibiotic shortages as barriers. (4) Adaptations and lessons learned - most participants (9/12) described late-2021 adaptations like virtual consultations. Core ASP components, such as audit and feedback and prescription restrictions, were notably affected. The framework of major themes from qualitative analysis of ASP disruptions is detailed in Table 2, with supporting subthemes in Tables 3-5.

**Table 2:** Framework of Major Themes from Qualitative Analysis of ASP Disruptions in Qazvin, Iran

Theme	Description	Example Quote
Empirical Prescribing	Increased use of antibiotics without confirmed bacterial infections, driven by diagnostic uncertainty and external pressures.	“The lack of rapid diagnostics led to more empirical prescribing” (Physician, P3)
Stewardship Disruption	Suspension of ASP activities, such as audits and consultations, due to staff redeployment and resource reallocation.	“The infectious diseases team was managing COVID units. ASP meetings stopped completely” (Infectious Disease Specialist, P1)
Systemic Constraints	Barriers including laboratory delays, antibiotic shortages, and limited resources hindering effective ASP implementation.	“Shortages forced us to use whatever antibiotics were available, even if not ideal” (Clinical Pharmacist, CP1)
Adaptive Strategies	Implementation of low-cost solutions, such as virtual consultations, to mitigate ASP disruptions in resource-limited settings.	“Social media posts about antibiotics curing COVID-19 pushed us to prescribe more than we wanted” (Physician, P9) (reflecting pressure countered by virtual consultations)

Theme one: increased empirical prescribing

All participants reported a shift to empirical prescribing during the pandemic’s peak, driven by diagnostic uncertainty, fear of superinfections, and public pressure. Broad-spectrum antibiotics like ceftriaxone and meropenem were used preemptively. “In the early COVID waves, nearly every patient with respiratory symptoms was started on antibiotics-mostly ceftriaxone or azithromycin-regardless of infection signs” (Pharmacist, P10); “We were terrified of missing something, so we gave meropenem or vancomycin right away” (Pulmonologist, P7). The return to rational prescribing by late 2021 was reported by participants, though variability persisted in emergency settings due to ongoing high patient volumes, reflecting perceptions rather than verified outcomes. Detailed subcodes for this theme are presented in Table 3.

**Table 3:** Thematic Coding of Antibiotic Prescription Across Pandemic Phases

Time Period	Mid-Level Themes	Subcodes
Before the Pandemic	Rational prescribing patterns	Prescribing based on clinical indications
		Limited use of broad-spectrum antibiotics
During the Pandemic	Increased empirical prescribing	Multiple antibiotics for COVID-19 patients without bacterial signs
		Fear-driven decision-making
		Anxiety about patient mortality, public pressure
After the Pandemic	Return to rational prescribing	Re-adoption of first-line treatments, guideline adherence

Theme two: disruption of antimicrobial stewardship programs

The COVID-19 pandemic disrupted antimicrobial stewardship programs (ASPs) in two teaching hospitals in Qazvin, Iran, halting key activities like audits and approval protocols due to staff redeployment to COVID-19 care. One participant stated, “The infectious diseases team was managing COVID units. ASP meetings stopped completely” (Infectious Disease Specialist, P1). Disruptions, reported by 10/12 participants, lasted through mid-2021, with informal practices like ad hoc specialist consultations used as temporary workarounds. Subthemes, detailed in Table 4, include prescribing without indication, common in emergency settings, and limited stewardship training, leading to reliance on past experiences.

**Table 4:** Disruptions in Antimicrobial Stewardship Programs

Main Theme	Subtheme	Description
Pandemic-Driven Overprescribing	Prescribing Without Indication	Antibiotics prescribed without confirmed bacterial infections
	ER-Based Empirical Prescribing	Common in emergency settings without adequate testing
Educational Gaps	Lack of Stewardship Training	Limited formal education on rational antibiotic use
	Reliance on Experience	Prescribers relied on past experiences due to lack of guidance

Theme three: external and systemic influences on prescribing

External factors, particularly media-driven public misconceptions, and systemic constraints, such as laboratory delays and antibiotic shortages, further undermined AMS efforts in Qazvin’s hospitals. Participants reported that media coverage and misinformation about antibiotics’ efficacy against COVID-19 increased patient demands, with one noting, “Patients demanded antibiotics after seeing news reports claiming they could prevent severe COVID, which pressured us to prescribe empirically” (Physician, P5), and another stating, “Social media spread myths about azithromycin curing COVID, leading to overuse” (Clinical Pharmacist, CP1). Systemic barriers included delayed diagnostics, as one participant highlighted, “The lack of rapid diagnostics led to more empirical prescribing during the first wave” (Physician, P3), and “Blood culture results were delayed-patients were discharged or deteriorated before we got them” (Lab Supervisor, P9). Recovery efforts, such as reinstating audit and feedback by late 2021, were reported by participants as implemented weekly but were inconsistent due to staffing shortages, reflecting perceptions rather than measured outcomes. Subthemes, detailed in Table 5, include media-driven demand, lack of real-time data, stock-driven prescribing, and fragmented oversight.

**Table 5:** External and Systemic Influences on Antibiotic Prescribing

Main Theme	Subtheme	Description
External Influences	Media and Public Messaging	Misinformation drove inappropriate antibiotic demand
Technology Limitations	Lack of Real-Time Data Access	Unavailable microbiological data hindered evidence-based prescribing
Systemic Influences	Stock-Driven Prescribing	Antibiotic choices based on availability, not clinical need
	Financial Constraints	Cost concerns impacted prescribing decisions
Organizational Challenges	Fragmented Oversight	Inconsistent prescribing due to lack of centralized supervision
Recovery Efforts	Audit and Feedback	Periodic audits improved prescribing behavior

Theme four: adaptations and lessons learned

Most participants (9/12) described adaptive measures by late 2021, such as virtual consultations. “We restarted weekly case reviews virtually-it brought back control over antibiotic prescribing practices” (Infectious Disease Specialist, P2). A participant noted, “Virtual reviews helped us maintain oversight despite staff shortages” (Clinical Pharmacist, CP1), indicating the role of technology in sustaining AMS during the pandemic. Virtual consultations were reported as widely used by infectious disease specialists and pharmacists but less consistently by residents, with no formal evaluation of their sustainability or long-term impact.

## Discussion

The study’s objectives-to explore healthcare providers’ perceptions of prescribing changes, ASP disruptions, and adaptations-were addressed through four themes reflecting participant-reported experiences: increased empirical prescribing, stewardship disruptions, systemic constraints, and adaptations. These findings provide context-specific insights into AMS challenges in two Qazvin hospitals, though limited to perceptions and requiring further validation. The findings, derived from 12 interviews in two Qazvin hospitals, highlight local challenges, with international comparisons providing broader context.

Disruption of antimicrobial stewardship programs

The COVID-19 pandemic disrupted ASPs in two teaching hospitals in Qazvin, Iran, halting critical activities like audits and approval protocols due to staff redeployment to COVID-19 care, as participants reported (e.g., “The infectious diseases team was managing COVID units. ASP meetings stopped completely” (Infectious Disease Specialist, P1). This led to increased empirical prescribing without confirmed bacterial infections, exacerbated by limited stewardship training and reliance on past experience, mirroring global challenges in maintaining AMS during pandemics [9]. In the UK, 64% of respondents reported increased broad-spectrum antibiotic use due to disrupted oversight, mitigated by procalcitonin tests, which were unavailable in Qazvin due to resource constraints, highlighting disparities in diagnostic capacity [9].

External and systemic influences on prescribing

Media-driven public misconceptions and systemic barriers, including laboratory delays and antibiotic shortages, intensified inappropriate prescribing in Qazvin’s hospitals, aligning with global trends [10-14]. Participants noted that misinformation about antibiotics’ efficacy against COVID-19 fueled patient demands, with one stating, “Patients demanded antibiotics after seeing news reports claiming they could prevent severe COVID” (Physician, P5), consistent with global reports of misinformation-driven antibiotic overuse [12, 13]. Systemic constraints, such as delayed diagnostics (e.g., “The lack of rapid diagnostics led to more empirical prescribing” (Physician, P3), mirrored India’s issues with unreliable antibiograms [11]. No specific measures to improve lab turnaround times were reported, though antibiotic shortages were partially mitigated by late 2021 through supply chain adjustments, as per participant reports. Sociocultural pressures, such as public demand for antibiotics, remained unaddressed, underscoring the need for targeted education campaigns. International responses varied: the UAE implemented national guidelines [10], while India used focus groups [11]. Qazvin’s reliance on virtual consultations, reported as adopted by late 2021, reflects a resource-driven adaptation, though their inconsistent use and lack of evaluation limit insights into their impact, unlike the UAE’s structured digital tools [10]. A US study highlighted similar overuse, with 71% of COVID-19 patients receiving antibiotics despite only 4% having bacterial coinfections [14]. Public education campaigns and improved laboratory capacity are essential to counter misinformation and strengthen AMS in settings like Iran.

ASP disruptions and responses in high-income countries

The COVID-19 pandemic disrupted ASPs in Qazvin, halting audits and approval protocols due to staff redeployment (e.g., “The infectious diseases team was managing COVID units. ASP meetings stopped completely” (Infectious Disease Specialist, P1), mirroring challenges in high-income countries [9, 14]. In the UK, 64% of respondents reported increased broad-spectrum antibiotic use, mitigated by procalcitonin tests, unavailable in Qazvin, where resource constraints led to reliance on informal consultations [9]. Similarly, a US study found 71% of COVID-19 patients received antibiotics despite only 4% having bacterial coinfections, reflecting diagnostic uncertainty [14]. High-income countries leveraged technologies like procalcitonin testing and virtual ward rounds, unlike Qazvin, highlighting resource disparities.

Media, systemic barriers, and adaptations in low- and middle-income countries (LMICs)

Reflecting local experiences in Qazvin, ASP disruptions were compounded by media-driven misinformation, laboratory delays, and antibiotic shortages, intensifying empirical prescribing, potentially similar to challenges in other Iranian or LMIC settings [10-13]. Participants reported patient demands fueled by misconceptions (e.g., “Patients demanded antibiotics or antiviral drugs (e.g., azithromycin, ceftriaxone) after seeing news reports” (Physician, P5). Lessons from the UAE (e.g., digital tools) and India (e.g., focus groups) suggest Iran could explore virtual consultations and community education, though infrastructure limitations hinder rapid diagnostic adoption [10, 11]. Qazvin’s virtual consultations, reported as adopted by late 2021, were used inconsistently and remain unevaluated, unlike Scotland’s structured interprofessional collaboration [15, 16]. Public education campaigns and improved laboratory capacity are essential to mitigate misinformation and strengthen AMS in resource-limited settings.

Implications for policy and practice

This study addresses a critical public health issue by examining participant-reported disruptions to ASPs during the COVID-19 pandemic in Qazvin, Iran, offering exploratory insights for strengthening AMS in resource-limited settings. Exploratory suggestions include virtual case reviews and public education campaigns to counter misinformation, pending further research to assess feasibility in Iran’s resource-limited context. Hospital antibiogram monitoring could track AMR trends, though no such efforts were reported in the studied hospitals. Barriers, such as limited funding for diagnostics and training gaps, require targeted investment. Additional strategies involve enhancing diagnostic capacity, improving antibiotic supply chains, and integrating AMS training into medical curricula, subject to validation.

Limitations and strengths

The small sample size (n=12) and limited representation of non-physician roles (e.g., one pharmacist, one lab supervisor) restrict the diversity of perspectives, potentially missing broader AMS dynamics. The absence of quantitative triangulation (e.g., antibiotic usage or laboratory data) limits validation of reported prescribing changes or stewardship recovery, restricting findings to participant perceptions. Self-reported data may be subject to recall or social desirability bias. The single-center focus on two Qazvin hospitals limits the applicability of findings to other Iranian or LMIC contexts, and findings should not be overgeneralized. The qualitative design precludes causal inferences, and institutional hierarchy may have influenced responses. Strengths include the study’s timely focus, the phenomenological approach capturing in-depth experiences, and purposive sampling until thematic saturation, achieved after the 10th interview. The diverse participant pool provides multifaceted insights, and international contextualization enhances robustness. Adherence to the COREQ checklist [8], supported by triangulation, member checking, and an audit trail, strengthens methodological credibility. Future mixed-methods research should quantify prescribing changes and validate adaptive strategies’ feasibility across diverse settings.

## Conclusions

This qualitative study provides context-specific insights into participant-reported disruptions and adaptations in AMS in two Qazvin hospitals during the COVID-19 pandemic. Increased empirical prescribing was reported due to diagnostic uncertainty and resource constraints, with adaptations like virtual consultations noted, though their sustainability remains unevaluated. International comparisons highlight shared challenges and the need for context-specific protocols and diagnostics. Findings are limited to participant perceptions and require further validation through mixed-methods research. Exploratory strategies, such as virtual tools and public education, offer potential for resource-limited settings like Iran, pending feasibility studies to ensure antibiotic efficacy in future crises.

## Appendices

Appendix 1

Interview Guide

1. Participant Information Sheet

The information provided below will help you understand why we are conducting this study and what it is about. Please read the information below and feel free to ask for clarification or additional information. We thank you for taking the time to read this.

What is the purpose of the project? Rational use of antibiotics helps prevent the emergence of antimicrobial resistance, improves patient healthcare outcomes, and reduces healthcare costs. The rational use of antibiotics faced significant challenges during the COVID-19 pandemic. The nature and impact of behavioral changes in physicians’ antibiotic prescribing practices during and after the COVID-19 pandemic remain unclear.

Main Objectives This study aims to investigate the impact of COVID-19 on antibiotic prescribing practices in two hospitals, Bu-Ali Sina and Velayat, affiliated with Qazvin University of Medical Sciences. It also explores the perceptions of physicians involved in treating hospitalized COVID-19 patients regarding the effectiveness of antibiotic prescribing, optimization of antimicrobial use, and whether these strategies can be adopted in hospitals during potential future waves of COVID-19 or other pandemics.

Secondary Objectives

To examine the strategies used for antibiotic prescribing in hospitals before COVID-19 and the opinions of healthcare teams involved in treating COVID-19 patients regarding the effectiveness of these strategies.

To investigate structural changes caused by COVID-19 (e.g., use of antibiotic prescribing forms, implementation of COVID-19 patient management guidelines, etc.) and their impact on antibiotic prescribing practices in hospitals.

To discuss barriers and facilitators in providing rational antibiotic prescribing services during the COVID-19 pandemic.

To explore any new or different activities adopted in hospitals to address challenges in antibiotic prescribing and the opinions of physicians regarding the effectiveness of these activities.

Why am I conducting this project? This study is being conducted to help understand the impact of COVID-19 on antibiotic prescribing activities in hospitals in the UK. The outcomes of this study will highlight what new strategies have been implemented and the perceptions of antibiotic prescribing teams regarding their effectiveness in optimizing antimicrobial use, as well as whether these strategies can be adopted in hospitals during ongoing or future pandemics.

Do I have to participate? Your participation in this study is entirely voluntary, and there is no obligation to participate. However, your participation is crucial to achieving the objectives outlined above, so we encourage you to participate.

What do I need to do if I agree to participate? You are invited to participate in an interview that will take approximately 45 to 60 minutes of your time. The interview will be audio-recorded. Discussions during the interviews will be recorded and transcribed by the project coordination team. One year after transcription, the recordings will be deleted. Only the research team will have access to this data. All collected data will be anonymized and will not include participant identifiers.

What are the benefits of participating? Your participation will help achieve the study’s objectives. Participation in this study will help identify rational antibiotic prescribing activities that have been effective in optimizing the use of this drug class and whether these activities can be implemented in hospitals during potential future waves of COVID-19 or other pandemics. It is anticipated that the overall results of this study will contribute to improved antimicrobial prescribing oversight in hospitals through policy and activity development.

Are there any disadvantages to participating? Other than the time required, there are no foreseeable disadvantages to your participation. At any stage of the process, you can request additional information or speak directly with the study team (contact details provided below).

Will all my information be kept confidential? All collected information will be stored on a secure computer. Discussions during the interviews will be recorded and transcribed by the project coordination team. One year after transcription, the recordings will be deleted. Only the research team will have access to this data. All collected data will be anonymized and will not include participant identifiers. It will not be possible to link any specific comment to any participant.

What happens when the study is complete? The interview transcripts will be analyzed and prepared for the final manuscript for publication. It is anticipated that the overall results of this study will contribute to improved antimicrobial prescribing oversight in hospitals through policy and activity development.

What will happen to the study results? The study results will be shared in the form of an article and published in scientific journals and conferences.

Who has reviewed and approved the study, and who can I contact for more information? This study has been reviewed and approved by Qazvin University of Medical Sciences.

2. Consent Form

How old are you? [] 18-29 years [] 30-39 years [] 40-49 years [] 50-59 years [] 60 years and above

What is your gender? [] Female [] Male [] Prefer not to disclose

Please check the boxes to confirm your agreement with the following statements:

I have read and understood the information about the project as provided in the Participant Information Sheet. ☐

I have been given the opportunity to ask questions about the project and my participation, and I have received answers to any questions I raised. ☐

I voluntarily agree to participate in this project. ☐

I understand that I can withdraw at any time during the interview without providing a reason, and I will not be penalized or questioned about my withdrawal. ☐

The procedures regarding confidentiality have been clearly explained to me. ☐

I understand that discussions during the interviews will be recorded and transcribed by the project coordination team, and I know that one year after transcription, the recordings will be deleted. ☐

I understand that any comments I make during the interview discussions cannot be linked to me but may be quoted anonymously in reports/publications. The use of data in research, publications, sharing, and archiving has been explained to me. ☐

I understand that the data will be accessible to the project coordinator. ☐

Study Coordinator: Dr. Abbas Allami Participant’s Name, Signature, and Date:

Interview Guide This interview will take approximately 45 minutes. Explain the confidentiality of the data for verbal consent:

All collected information will be stored on a secure computer.

Recorded discussions during the interviews will be transcribed by the project coordination team and deleted one year after transcription.

All collected data will be anonymized and will not include participant identifiers.

It will not be possible to link any specific comment to any participant.

Only the research team will have access to this data.

Baseline Questionnaire To begin, we have a few questions about participants to indicate which applies:

What is your current role in antibiotic prescribing? []

How many years have you worked in this role? [] Less than 2 years [] 2 to 5 years [] 6 to 9 years [] 10 years or more

Questions:

Pre-COVID-19 Conditions

What were the antimicrobial stewardship strategies/activities in your hospital?

Do you think these activities were effective? If yes, why? If not, why not?

Can you share an example of a successful antibiotic prescribing activity that you or your team introduced?

Can you tell me who you think are the key stakeholders in implementing antimicrobial stewardship strategies in your hospital? Why?

Do you think you (or others) were confident that the hospital supported the implementation of these strategies? If yes, why? If not, why not?

Do you think you (or others) were committed to implementing these strategies? If yes, why? If not, why not?

Conditions During and After the Pandemic 7. Do you think you received sufficient information from institutions/government authorities regarding the implementation of rational antibiotic prescribing activities? If yes, why? If not, why not? 8. Have you encountered structural changes caused by COVID-19 (e.g., use of antibiotic prescribing forms, implementation of COVID-19 patient management guidelines, etc.) and their impact on antibiotic prescribing activities in hospitals? If yes, can you explain these structural changes and how they affected antibiotic prescribing? 9. To what extent did these structural changes facilitate or hinder the implementation of rational antibiotic prescribing activities? 10. To what extent do you think the COVID-19 pandemic hindered the implementation of routine stewardship activities for controlling excessive antimicrobial prescribing? 11. What challenges arose in the use of antibiotics? Why? 12. Which antibiotic prescribing activities were most affected? In what ways? 13. Do you feel that COVID-19 had a positive (or negative) impact on antibiotic prescribing practices? If yes, why? If not, why not? 14. What are the positive and negative impacts of social media on antibiotic prescribing activities in your setting? 15. Can you share an example of the role of pharmaceutical companies and their advertisements in antibiotic prescribing (especially new antibiotics) introduced to you or your team? 16. Do you see COVID-19 as an opportunity for more rational antibiotic prescribing and curbing irrational prescribing? 17. To what extent did these strategies impact the treatment of COVID-19 patients? 18. Based on your experience with rational antibiotic prescribing during the pandemic, what other strategies can we adopt for use in hospitals? 19. To what extent did clinical guidelines influence your behavior in rational antibiotic prescribing during the COVID-19 pandemic? 20. What lessons have you learned from the COVID-19 pandemic, and how can this learning be useful in future pandemics? 21. Are there any other factors we haven’t addressed that you think might be important? 22. Is there anything else you’d like to add? 23. Do you have any further questions?
